# Synthesis and crystal structure of a 6-chloro­nicotinate salt of a one-dimensional cationic nickel(II) coordination polymer with 4,4′-bi­pyridine

**DOI:** 10.1107/S2056989020004193

**Published:** 2020-04-02

**Authors:** Nives Politeo, Mateja Pisačić, Marijana Đaković, Vesna Sokol, Boris-Marko Kukovec

**Affiliations:** a Department of Physical Chemistry, Faculty of Chemistry and Technology, University of Split, Ruđera Boškovića 35, HR-21000 Split, Croatia; b Department of Chemistry, Faculty of Science, University of Zagreb, Horvatovac, 102a, HR-10000 Zagreb, Croatia

**Keywords:** nickel(II), 6-chloro­nicotinic acid, 4,4′-bi­pyridine, coordination polymer, hydrogen-bond motif, crystal structure

## Abstract

A 6-chloro­nicotinate (6-Clnic) salt of a one-dimensional cationic nickel(II) coordination polymer with 4,4′-bi­pyridine (4,4′-bpy), namely, *catena*-poly[[[tetra­aqua­nickel(II)]-μ-4,4′-bi­pyridine-κ^2^ *N*:*N*′] bis­(6-chloro­nicotinate) tetra­hydrate], {[Ni(C_10_H_8_N_2_)(H_2_O)_4_](C_6_H_3_ClNO_2_)_2_·4H_2_O}_*n*_ or {[Ni(4,4′-bpy)(H_2_O)_4_](6-Clnic)_2_·4H_2_O}_*n*_, (**1**), was prepared by the reaction of nickel(II) sulfate hepta­hydrate, 6-chloro­nicotinic acid and 4,4′-bi­pyridine in a mixture of water and ethanol. The mol­ecular structure of **1** comprises a one-dimensional polymeric {[Ni(4,4′-bpy)(H_2_O)_4_]^2+^}_*n*_ cation, two 6-chloro­nicotinate anions and four water mol­ecules of crystallization per repeating polymeric unit. The nickel(II) ion in the polymeric cation is octa­hedrally coordinated by four water mol­ecule O atoms and by two 4,4′-bi­pyridine N atoms in the *trans* position. The 4,4′-bi­pyridine ligands act as bridges and, thus, connect the symmetry-related nickel(II) ions into an infinite one-dimensional polymeric chain extending along the *b-*axis direction. In the extended structure of **1**, the polymeric chains of {[Ni(4,4′-bpy)(H_2_O)_4_]^2+^}_*n*_, the 6-chloro­nicotinate anions and the water mol­ecules of crystallization are assembled into an infinite three-dimensional hydrogen-bonded network *via* strong O—H⋯O and O—H⋯N hydrogen bonds, leading to the formation of the representative hydrogen-bonded ring motifs: tetra­meric *R* ^2^_4_(8) and *R* ^4^_4_(10) loops, a dimeric *R* ^2^_2_(8) loop and a penta­meric *R* ^4^_5_(16) loop.

## Abstract 2

The title compound is a 6-chloro­nicotinate salt of a one-dimensional cationic nickel(II) coordination polymer with 4,4′-bi­pyridine. The nickel(II) ion in the polymeric cation is octa­hedrally coordinated by four water mol­ecule O atoms and by two 4,4′-bi­pyridine N atoms. The 4,4′-bi­pyridine ligands act as bridges, connecting the symmetry-related nickel(II) ions into polymeric chains along the *b-*axis direction. In the extended structure, these chains, the anions and the water mol­ecules of crystallization are assembled into a three-dimensional network *via* strong O—H⋯O and O—H⋯N hydrogen bonds

## Chemical context   

Functional coordination polymers have attracted great inter­est in recent years, mostly due to their aesthetics and many inter­esting properties such as catalytic, magnetic and luminescent, potential for use in gas storage and separation, mol­ecular sensing (Mueller *et al.*, 2006 ▸; Bosch *et al.*, 2017 ▸; Zhang *et al.*, 2015 ▸; Zeng *et al.*, 2014 ▸, 2016 ▸; Douvali *et al.*, 2015 ▸; Xu *et al.*, 2017 ▸; Zhou *et al.*, 2017 ▸).

The organic ligands, used as building blocks in the construction of coordination polymers, need to be multifunctional, which is evident from the position, coordination ability and steric hindrance of their donor atoms and/or groups. The design of functional coordination polymers with the desired structures is not always straightforward and is strongly dependent on the experimental conditions including the type of solvents, starting metal salts, additional ligands, temperature, hydro­thermal conditions and pH value (Li *et al.*, 2016 ▸; Zhou *et al.*, 2016 ▸; Gu *et al.*, 2016 ▸). Aromatic carb­oxy­lic acids with additional functional groups have become popular in the design of coordination polymers. The main reasons are the many possible and unpredictable coordination modes of this type of ligand and their affinity for participation in supra­molecular inter­actions (Gu *et al.*, 2016 ▸, 2017 ▸, 2018 ▸; Wang *et al.*, 2016 ▸; Zhang *et al.*, 2019 ▸).

The metal complexes of chlorinated analogues of the nicotinate anion (*e.g.* 2-chloro­nicotinate and 5-chloro­nicotinate) have not been particularly well-studied [as of March 2020, there are around 20 crystal structures in the CSD (Groom *et al.*, 2016 ▸) for each ligand]. Furthermore, no metal complexes of the 4-chloro­nicotinate anion have been reported. The crystal structures of only three metal complexes of 6-chloro­nicotinate (6-Clnic) are known so far (Xia *et al.*, 2012*a* ▸,*b* ▸; Li *et al.*, 2006 ▸). Recently, we have reported the synthesis, crystal structure and properties of a one-dimensional nickel(II) coordination polymer with mixed ligands: 6-fluoro­nicotinate as the main ligand and 4,4′-bi­pyridine (4,4′-bpy) as the supporting ligand (Politeo *et al.*, 2020 ▸).
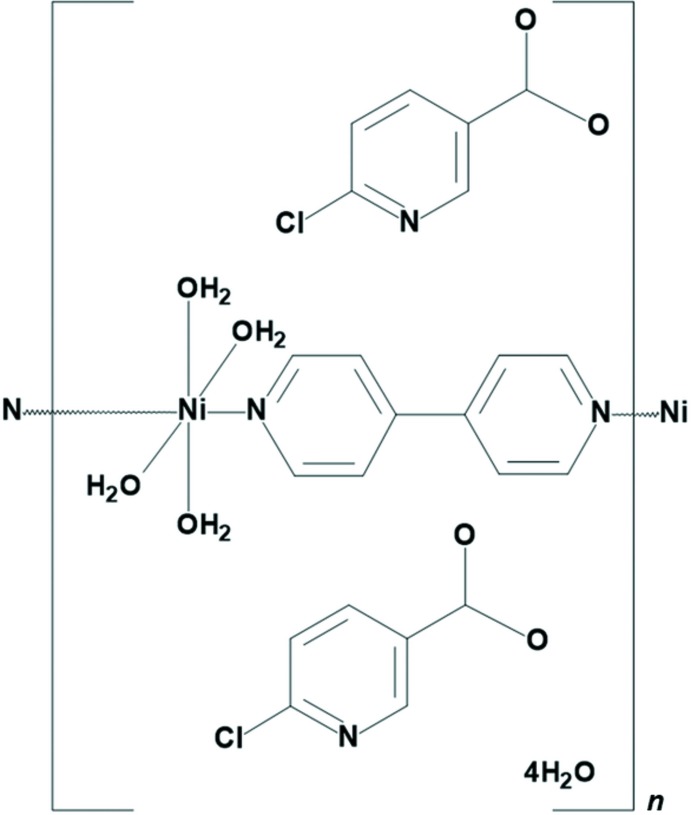


In a continuation of our work on coordination polymers with mixed ligands, we set out to prepare a similar coordination polymer with 6-chlorinicotinate and 4,4′-bi­pyridine, as we did with 6-fluoro­nicotinate (Politeo *et al.*, 2020 ▸). Therefore, we carried out the synthesis and crystallization under the same experimental conditions (in a mixture of water and ethanol and with the same molar ratios of the nickel(II) sulfate and ligands), in hope that the analogous nickel(II) coordination polymer could be obtained. We also wanted to examine the influence of the possible weak inter­molecular inter­actions involving the chlorine atoms (*e.g.* C—H⋯Cl inter­actions) on the assembly of the polymeric chains in the crystal packing, especially since the analogous C—H⋯F inter­actions were not found in the crystal packing of the nickel(II) coordination polymer with 6-fluoro­nicotinate (Politeo *et al.*, 2020 ▸). Unfortunately, we were not able to prepare the desired nickel(II) coordination polymer under these experimental conditions, but instead we obtained a 6-chloro­nicotinate salt of a one-dimensional cationic nickel(II) coordination polymer with 4,4′-bi­pyridine, namely the title compound, {[Ni(4,4′-bpy)(H_2_O)_4_](6-Clnic)_2_·4H_2_O}_*n*_, (**1**).

## Structural commentary   

**Figure 1:**
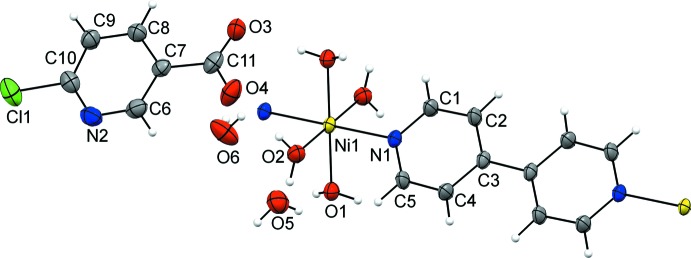
The mol­ecular structure of **1**, comprising a {[Ni(4,4′-bpy)(H_2_O)_4_]^2+^}_*n*_ cation, 6-chloro­nicotinate anion and water mol­ecules of crystallization. The atomic numbering scheme of the asymmetric unit is shown and displacement ellipsoids are drawn at the 40% probability level.

**Figure 2:**
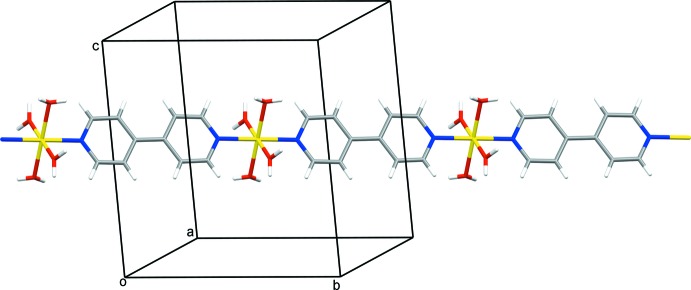
The infinite one-dimensional polymeric chain of {[Ni(4,4′-bpy)(H_2_O)_4_]^2+^}_*n*_ cations in **1**, extending along the *b*-axis direction.

As the nickel(II) ion is situated on an inversion center, the asymmetric unit of **1** contains one half of a nickel(II) ion, two coordinated water mol­ecules, one 6-chloro­nicotinate ligand, one half of a 4,4′-bi­pyridine ligand and two water mol­ecules of crystallization (Fig. 1 ▸). Therefore, the mol­ecular structure of **1** comprises a one-dimensional polymeric {[Ni(4,4′-bpy)(H_2_O)_4_]^2+^}_*n*_ cation and two 6-chloro­nicotinate anions and four uncoordinated water mol­ecules per repeating polymeric unit. The nickel(II) ion in the polymeric {[Ni(4,4′-bpy)(H_2_O)_4_]^2+^}_*n*_ cation is octa­hedrally coordinated by four water mol­ecule O atoms (O1, O2, O1^i^ and O2^i^) [symmetry code: (i) −*x* + 1, −*y* + 1, −*z* + 1] and by two 4,4′-bi­pyridine N atoms (N1 and N1^i^) in the *trans* position (N1^i^—Ni1—N1 = 180°). The 4,4′-bi­pyridine ligands act as bridges and, thus, connect the symmetry-related nickel(II) ions into infinite one-dimensional polymeric chains extending along the *b*-axis direction (Fig. 2 ▸).

The octa­hedral coordination environment around the nickel(II) ion is only slightly distorted, as indicated by the angles for the *cis* pairs of the ligating atoms [89.00 (5)–91.00 (5)°]. The Ni1—O1 and Ni1—O2 bond lengths [2.0643 (15) Å and 2.0850 (13) Å, respectively] are very similar to each other and comparable to those seen in the related structures containing {[Ni(4,4′-bpy)(H_2_O)_4_]^2+^}_*n*_ cation. The Ni—N1 bond length [2.0715 (14) Å] is also in agreement with those reported for the structures containing the {[Ni(4,4′-bpy)(H_2_O)_4_]^2+^}_*n*_ cation (Zheng *et al.*, 2002 ▸; Gong *et al.*, 2009 ▸; Li, 2011 ▸; Gao *et al.*, 2016 ▸; Sun *et al.*, 2013 ▸; Wang *et al.*, 2006 ▸; Sanram *et al.*, 2016 ▸; Hu & Zhang, 2010 ▸).

The 4,4′-bypyridine ring is not coplanar with either coord­inated water mol­ecule O1 or O2 atoms and is rotated about the Ni1—N1 bond (by approximately 2°), as is evident from the torsion angles Ni1—N1—C5—C4 and Ni1—N1—C1—C2 [177.75 (16) and −177.83 (16)°, respectively].

## Supra­molecular features   

**Figure 3:**
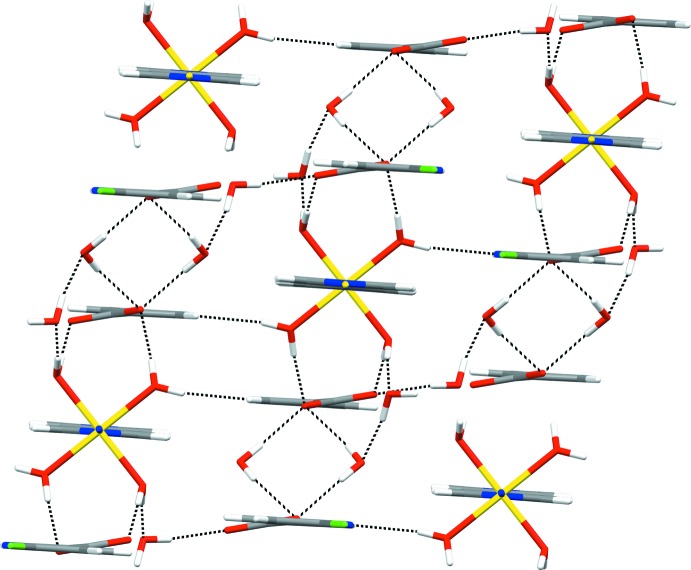
A fragment of the infinite hydrogen-bonded network of **1** viewed along the *b*-axis direction. The polymeric chains of {[Ni(4,4′-bpy)(H_2_O)_4_]^2+^}_*n*_ (represented as monomeric mol­ecules in this projection), 6-chloro­nicotinate anions and water mol­ecules of crystallization are connected by O—H⋯O and O—H⋯N hydrogen bonds (represented by dotted lines) within the hydrogen-bonded framework.

The extended structure of **1** features strong O—H⋯O and O—H⋯N hydrogen bonds, weak C—H⋯O hydrogen bonds (Table 1 ▸) and π–π inter­actions [*Cg*2⋯*Cg*2; where *Cg*2 is the centroid of the 6-chloro­nicotinate pyridine ring N2/C6–C10; *Cg*2⋯*Cg*2 distance = 3.6769 (12) Å; dihedral angle between the planes = 0.00 (10)°; slippage = 1.085 Å]. The strong hydrogen bonds link the polymeric chains of {[Ni(4,4′-bpy)(H_2_O)_4_]^2+^}_*n*_, the 6-chloro­nicotinate anions and the water mol­ecules of crystallization into an infinite three-dimensional network. The structure can be better analyzed if viewed down the *b-*axis direction (the direction along which the polymeric chain of {[Ni(4,4′-bpy)(H_2_O)_4_]^2+^}_*n*_ runs). In that projection, the polymeric chains can be regarded as monomeric mol­ecules that are inter­connected with the 6-chloro­nicotinate anions and water mol­ecules of crystallization into a hydrogen-bonded framework (Fig. 3 ▸). The polymeric chains are exclusively hydrogen-bonded to 6-chloro­nicotinate anions and water mol­ecules, whilst the 6-chloro­nicotinate anions are additionally assembled by π–π inter­actions between symmetry-related 6-chloro­nicotinate pyridine rings.

**Figure 4:**
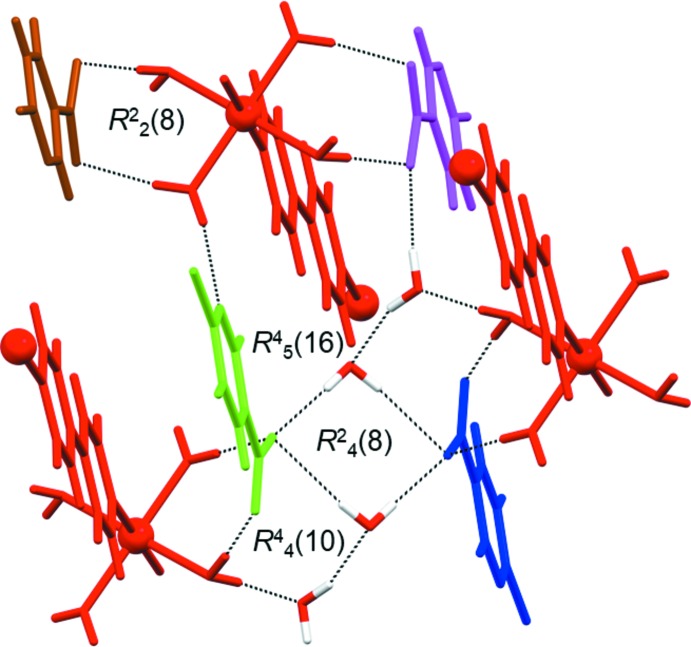
The representative hydrogen-bonded ring motifs (shown by dotted lines) found within the hydrogen-bonded framework of **1**, *viz*. the tetra­meric 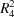
(8) and 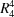
(10) motifs, a dimeric 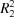
(8) motif and a penta­meric 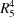
(16) motif. The polymeric chains of {[Ni(4,4′-bpy)(H_2_O)_4_]^2+^}_*n*_ are represented as momomeric mol­ecules and shown in red, and various symmetry-related 6-chloro­nicotinate anions are shown in brown, green, blue and pink (see text).

There are some representative supra­molecular ring motifs within the hydrogen-bonded framework of **1**: tetra­meric 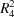
(8) and 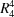
(10) motifs, a dimeric 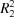
(8) motif and a penta­meric 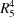
(16) motif (Fig. 4 ▸). The tetra­meric 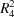
(8) motif is formed between two water mol­ecules of crystallization and two 6-chloro­nicotinate anions (indicated in blue and green); each 6-chloro­nicotinate anion is linked *via* a single carboxyl­ate O atom. The tetra­meric 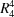
(10) motif is formed between the [Ni(4,4′-bpy)(H_2_O)_4_]^2+^}_*n*_ cation, a 6-chloro­nicotinate anion (indicated in red and green, respectively) and two water mol­ecules of crystallization; the cation participates in this motif *via* a coordinated water O atom and the 6-chloro­nicotinate anion *via* both carboxyl­ate O atoms. The dimeric 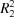
(8) motif is formed between the {[Ni(4,4′-bpy)(H_2_O)_4_]^2+^}_*n*_ cation and the 6-chloro­nicotinate anion (indicated in red and brown, respectively); the cation is involved in this motif *via* two coordinated water O atoms and the 6-chloro­nicotinate anion *via* both carboxyl­ate O atoms. Finally, the penta­meric 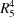
(16) motif is composed of the {[Ni(4,4′-bpy)(H_2_O)_4_]^2+^}_*n*_ cation, two 6-chloro­nicotinate anions (indicated in red, green and pink) and two water mol­ecules of crystallization; the cation participates in this motif *via* two coordinated water O atoms, one 6-chloro­nicotinate anion (shown in green) *via* both carboxyl­ate O atoms and the pyridine N atom and the other 6-chloro­nicotinate anion (shown in pink) *via* its carboxyl­ate O atom only (Fig. 4 ▸). Both coordinated water mol­ecules and water mol­ecules of crystallization participate in the formation of motifs as single- and double-proton donors [coordinated water mol­ecules as single-proton donors in the 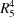
(16) and 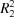
(8) motifs and double-proton donors in the 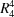
(10) motif only; water mol­ecules of crystallization as single-proton donors in the 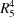
(16) motifs and 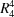
(10) motifs and double-proton donors in the 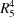
(16) and 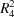
(8) motifs]. The water mol­ecules of crystallization also participate in some of these motifs [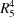
(16) and 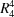
(10)] as single-proton acceptors. The 6-chloro­nicotinate pyridine N atoms act as single-proton acceptors in the 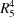
(16) motif only, whilst the carboxyl­ate O atoms act as both single- and double-proton acceptors [single in the 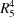
(16), 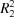
(8) and 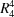
(10) motifs and double in the 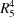
(16) and 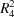
(8) motifs]. Two weak C—H⋯O inter­actions are also observed (Table 1 ▸).

There are no weak C—H⋯Cl inter­actions in the extended structure of **1**; we hoped that these inter­actions could have an impact on the assembly of the polymeric chains within the hydrogen-bonding framework of **1**: the polymeric chains do not contain the 6-chloro­nicotinate ligands, but the uncoord­inated 6-chloro­nicotinate anions could still participate in these inter­actions. However, the possible C—H⋯Cl inter­actions are most probably hindered by the extensive hydrogen bonding, involving strong O—H⋯O and O—H⋯N hydrogen bonds, which is reflected in the formation of various hydrogen-bonded motifs. This was expected because of the participation of the water mol­ecules of crystallization in the crystal packing of **1**, since the compound was crystallized from a mixed water–ethanol solution.

## Database survey   

Our aim in this work was to prepare a nickel(II) coordination polymer with the mixed ligands 6-chloro­nicotinate and 4,4′-bi­pyridine. However, we obtained a cationic nickel(II) coordination polymer with 4,4′-bi­pyridine, {[Ni(4,4′-bpy)(H_2_O)_4_]^2+^}*_n_.* The 6-chloro­nicotinate is not coordinated to the metal ion, but acts as a counter-ion. This was surprising, as we expected to obtain a coordination polymer similar to the one obtained with the closely related 6-fluoro­nicotinate anion under the same experimental conditions (Politeo *et al.*, 2020 ▸). The polymeric {[Ni(4,4′-bpy)(H_2_O)_4_]^2+^}_*n*_ cation is already well known from the literature, as it crystallizes with various carboxyl­ate anions such as fumarate (Zheng *et al.*, 2002 ▸), 3-[4-(carb­oxy­meth­oxy) phen­yl]propano­ate (Gong *et al.*, 2009 ▸), 3,3′-(*p*-phenyl­ene)diacrylate (Li, 2011 ▸), 2-carb­oxy-4-[4-(3-carb­oxy-4-carboxyl­atophen­oxy)phen­oxy]benzoate (Gao *et al.*, 2016 ▸), 3-(4-carb­oxy­phen­yl)propano­ate (Sun *et al.*, 2013 ▸), 1,2,4,5-benzene­tetra­carboxyl­ate (Wang *et al.*, 2006 ▸), 1,4-phenyl­enedi­propano­ate (Sanram *et al.*, 2016 ▸) and 2,3-naphthalenedi­carboxyl­ate (Hu & Zhang, 2010 ▸).

## PXRD and thermal analysis   

**Figure 5:**
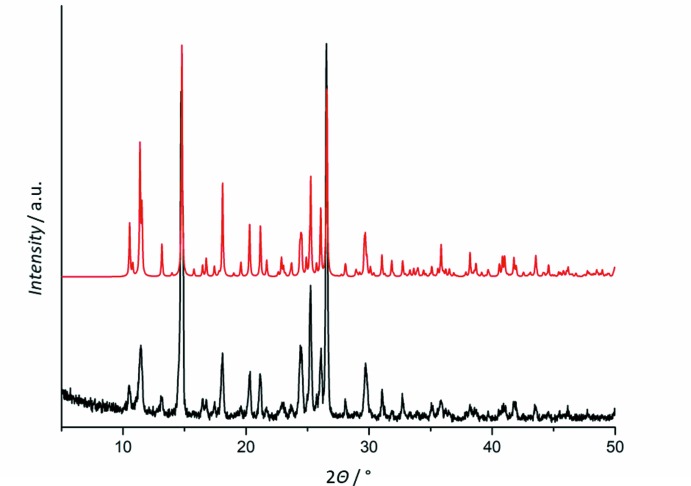
Experimental (bottom) and calculated (top) PXRD traces for **1**.

The experimental and calculated PXRD traces of **1** (Fig. 5 ▸) match nicely, indicating the phase purity of the bulk of **1**.

Compound **1** is thermally stable only up to 40°C (Fig. S1 in the supporting information). Both the coordinated (four) and uncoordinated (four) water mol­ecules were released in the same step (observed mass loss 20.3%, calculated 21.4%), with a pronounced endothermic peak in the DSC curve at 90°C. The thermal decomposition of **1** continues in a broad step (observed mass loss 55.2%) in the wide temperature range of 145–590°C (with two small peaks in the DSC curve at 216 and 480°C), which probably corresponds to the complete degradation of **1**. The remaining residue at 600°C is most probably NiO.

## Materials and methods   

All chemicals for the synthesis were purchased from commercial sources (Merck) and used as received without further purification. The IR spectrum was obtained in the range 4000–400 cm^−1^ on a Perkin–Elmer Spectrum Two^TM^ FTIR spectrometer in the ATR mode. The PXRD trace was recorded on a Philips PW 1850 diffractometer, Cu *K*α radiation, voltage 40 kV, current 40 mA, in the angle range 5–50° (2*θ*) with a step size of 0.02°. Simultaneous TGA/DSC measurements were performed at a heating rate of 10°C min^−1^ in the temperature range 25–600°C, under a nitro­gen flow of 50 ml min^−1^ on a Mettler–Toledo TGA/DSC 3+ instrument. Approximately 2 mg of sample was placed in a standard alumina crucible (70 µl).

## Synthesis and crystallization   

6-Chloro­nicotinic acid (0.0525 g, 0.3332 mmol) was dissolved in distilled water (5 ml) using an ultrasonic water bath, 4,4′-bi­pyridine (0.0244 g, 0.1562 mmol) was dissolved in ethanol (2 ml) and nickel(II) sulfate hepta­hydrate (0.0446 g, 0.1588 mmol) was dissolved in distilled water (2 ml). The solutions of the two ligands were first mixed together under stirring. The resulting solution was then slowly added to the nickel(II) sulfate solution under stirring. The pH of the final solution was adjusted to 7 by adding an ammonia solution dropwise. The obtained, clear solution was left to slowly evaporate at room temperature for approximately three weeks until light-green crystals of **1**, suitable for X-ray diffraction measurements, were obtained, which were collected by filtration, washed with their mother liquor and dried *in vacuo*. Yield: 0.0496 g (46%). Selected IR bands (ATR) (*ν*, cm^−1^): 3376 [*ν*(O—H)], 3078, 3059 [*ν*(C—H)], 1615 [*ν*(C=O)], 1579, 1539, 1419, 1388, 1360 [*ν*(C—C), *ν*(C—N)] (Fig. S2, Table S1 in the supporting information).

## Refinement   

Crystal data, data collection and structure refinement details are summarized in Table 2 ▸. C-bound H atoms were positioned geometrically and refined using riding model [C—H = 0.93 Å, *U*_iso_(H) = 1.2*U*_eq_(C) for the aromatic H atoms]. The H atoms belonging to the water mol­ecules were found in the difference-Fourier maps. The O—H distance was restrained to an average value of 0.82 Å using DFIX and DANG instructions. The isotropic *U*_iso_(H) values were also fixed [*U*_iso_(H) = 1.2*U*_eq_(O)].

The highest difference peak is 0.86 Å away from the O4 atom and the deepest difference hole is 0.84 Å away from the Cl1 atom.

## Supplementary Material

Crystal structure: contains datablock(s) I. DOI: 10.1107/S2056989020004193/hb7900sup1.cif

Structure factors: contains datablock(s) I. DOI: 10.1107/S2056989020004193/hb7900Isup2.hkl

IR, TGA and DSC data. DOI: 10.1107/S2056989020004193/hb7900sup3.docx

CCDC reference: 1992951

Additional supporting information:  crystallographic information; 3D view; checkCIF report

## Figures and Tables

**Table 1:** Hydrogen-bond geometry (Å, °)

*D*—H⋯*A*	*D*—H	H⋯*A*	*D*⋯*A*	*D*—H⋯*A*
O1—H11⋯O3^i^	0.81 (1)	1.95 (1)	2.756 (2)	175 (2)
O1—H12⋯O5	0.82 (1)	1.90 (1)	2.715 (2)	175 (2)
O2—H21⋯N2^ii^	0.81 (1)	2.08 (1)	2.885 (2)	172 (2)
O2—H22⋯O4	0.81 (1)	1.96 (1)	2.757 (2)	169 (2)
O5—H51⋯O3^iii^	0.82 (1)	1.96 (1)	2.776 (2)	172 (3)
O5—H52⋯O6^iv^	0.82 (1)	2.01 (1)	2.790 (3)	160 (3)
O6—H61⋯O4	0.82 (1)	1.94 (1)	2.753 (2)	177 (3)
O6—H62⋯O4^v^	0.81 (1)	2.23 (1)	3.035 (3)	174 (3)
C4—H4⋯O6^ii^	0.93	2.40	3.288 (3)	160
C9—H9⋯O5^vi^	0.93	2.53	3.447 (3)	169

Symmetry codes: (i) 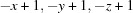; (ii) 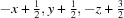; (iii) 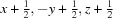; (iv) 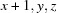; (v) 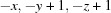; (vi) 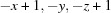.

**Table 2:** Experimental details

Crystal data	
Chemical formula	{[Ni(C_10_H_8_N_2_)(H_2_O)_4_](C_6_H_3_ClNO_2_)_2_·4H_2_O}_*n*_
*M*_r_	672.11
Crystal system, space group	Monoclinic, *P*2_1_/*n*
Temperature (K)	296
*a*, *b*, *c* (Å)	10.7997 (3), 11.2319 (2), 12.0225 (3)
β (°)	95.184 (2)
*V* (Å^3^)	1452.38 (6)
*Z*	2
Radiation type	Mo *K*α
μ (mm^−1^)	0.92
Crystal size (mm)	0.24 × 0.18 × 0.16

Data collection	
Diffractometer	Oxford Diffraction Xcalibur2 diffractometer with Sapphire 3 CCD detector
Absorption correction	Multi-scan (*CrysAlis PRO*; Rigaku OD, 2018 ▸)
*T*_min_, *T*_max_	0.927, 1.000
No. of measured, independent and observed [*I* > 2σ(*I*)] reflections	11778, 2541, 2144
*R*_int_	0.025
(sin θ/λ)_max_ (Å^−1^)	0.595

Refinement	
*R*[*F* ^2^ > 2σ(*F* ^2^)], *wR*(*F* ^2^), *S*	0.029, 0.074, 1.07
No. of reflections	2541
No. of parameters	211
No. of restraints	12
H-atom treatment	H atoms treated by a mixture of independent and constrained refinement
Δρ_max_, Δρ_min_ (e Å^−3^)	0.23, −0.23

Computer programs: *CrysAlis PRO* (Rigaku OD, 2018 ▸), *SHELXT* (Sheldrick, 2015*a* ▸), *SHELXL2018/3* (Sheldrick, 2015*b* ▸) and *Mercury* (Macrae *et al.*, 2020 ▸).

## supplementary crystallographic information

### Crystal data

**Table:**

[Ni(C_10_H_8_N_2_)(H_2_O)_4_](C_6_H_3_ClNO_2_)_2_·4H_2_O	*F*(000) = 696
*M**_r_* = 672.11	*D*_x_ = 1.537 Mg m^−^^3^
Monoclinic, *P*2_1_/*n*	Mo *K*α radiation, λ = 0.71073 Å
*a* = 10.7997 (3) Å	Cell parameters from 6296 reflections
*b* = 11.2319 (2) Å	θ = 4.4–32.2°
*c* = 12.0225 (3) Å	µ = 0.92 mm^−^^1^
β = 95.184 (2)°	*T* = 296 K
*V* = 1452.38 (6) Å^3^	Prism, light-green
*Z* = 2	0.24 × 0.18 × 0.16 mm

### Data collection

**Table:**

Oxford Diffraction Xcalibur2 diffractometer with Sapphire 3 CCD detector	2144 reflections with *I* > 2σ(*I*)
ω–scan	*R*_int_ = 0.025
Absorption correction: multi-scan (CrysAlisPro; Rigaku OD, 2018)	θ_max_ = 25.0°, θ_min_ = 4.2°
*T*_min_ = 0.927, *T*_max_ = 1.000	*h* = −12→12
11778 measured reflections	*k* = −13→13
2541 independent reflections	*l* = −14→14

### Refinement

**Table:**

Refinement on *F*^2^	Primary atom site location: dual
Least-squares matrix: full	Hydrogen site location: mixed
*R*[*F*^2^ > 2σ(*F*^2^)] = 0.029	H atoms treated by a mixture of independent and constrained refinement
*wR*(*F*^2^) = 0.074	*w* = 1/[σ^2^(*F*_o_^2^) + (0.0367*P*)^2^ + 0.3591*P*] where *P* = (*F*_o_^2^ + 2*F*_c_^2^)/3
*S* = 1.07	(Δ/σ)_max_ < 0.001
2541 reflections	Δρ_max_ = 0.23 e Å^−^^3^
211 parameters	Δρ_min_ = −0.23 e Å^−^^3^
12 restraints	

### Special details

**Table:**

Geometry. All esds (except the esd in the dihedral angle between two l.s. planes) are estimated using the full covariance matrix. The cell esds are taken into account individually in the estimation of esds in distances, angles and torsion angles; correlations between esds in cell parameters are only used when they are defined by crystal symmetry. An approximate (isotropic) treatment of cell esds is used for estimating esds involving l.s. planes.

### Fractional atomic coordinates and isotropic or equivalent isotropic displacement parameters (Å^2^)

**Table:**

	*x*	*y*	*z*	*U*_iso_*/*U*_eq_	
Ni1	0.500000	0.500000	0.500000	0.02724 (12)	
Cl1	0.14959 (7)	−0.20644 (6)	0.64253 (6)	0.0683 (2)	
N1	0.49579 (14)	0.68437 (13)	0.49747 (12)	0.0302 (4)	
N2	0.14853 (18)	0.02547 (18)	0.65643 (15)	0.0489 (5)	
O1	0.63069 (14)	0.50565 (11)	0.63625 (12)	0.0365 (3)	
H11	0.6796 (16)	0.5604 (14)	0.6359 (19)	0.044*	
H12	0.6766 (17)	0.4477 (13)	0.6434 (18)	0.044*	
O2	0.35666 (14)	0.50182 (12)	0.60512 (12)	0.0381 (3)	
H21	0.363 (2)	0.5067 (18)	0.6729 (8)	0.046*	
H22	0.2918 (13)	0.4706 (19)	0.5828 (17)	0.046*	
O3	0.21575 (15)	0.29848 (13)	0.36561 (13)	0.0513 (4)	
O4	0.15477 (15)	0.36711 (14)	0.52437 (14)	0.0577 (5)	
O5	0.77684 (18)	0.31004 (15)	0.67043 (15)	0.0578 (5)	
H51	0.765 (2)	0.280 (2)	0.7309 (13)	0.069*	
H52	0.8451 (14)	0.341 (2)	0.670 (2)	0.069*	
O6	−0.02489 (19)	0.4641 (2)	0.64545 (15)	0.0691 (5)	
H61	0.027 (2)	0.434 (2)	0.608 (2)	0.083*	
H62	−0.063 (2)	0.511 (2)	0.604 (2)	0.083*	
C1	0.5043 (2)	0.74728 (16)	0.40446 (16)	0.0366 (5)	
H1	0.509493	0.706173	0.337893	0.044*	
C2	0.5057 (2)	0.86964 (16)	0.40158 (16)	0.0368 (5)	
H2	0.511821	0.909144	0.334314	0.044*	
C3	0.49798 (17)	0.93401 (15)	0.49898 (15)	0.0280 (4)	
C4	0.48697 (19)	0.86865 (16)	0.59518 (16)	0.0361 (5)	
H4	0.480202	0.907506	0.662667	0.043*	
C5	0.48605 (19)	0.74622 (15)	0.59102 (16)	0.0359 (5)	
H5	0.478228	0.704445	0.656807	0.043*	
C6	0.1538 (2)	0.1347 (2)	0.61298 (18)	0.0456 (5)	
H6	0.147862	0.199385	0.660541	0.055*	
C7	0.16748 (18)	0.15756 (17)	0.50216 (16)	0.0356 (5)	
C8	0.1733 (2)	0.06049 (18)	0.43203 (17)	0.0414 (5)	
H8	0.180909	0.072105	0.356359	0.050*	
C9	0.1679 (2)	−0.05304 (19)	0.47386 (18)	0.0439 (5)	
H9	0.172347	−0.119476	0.428227	0.053*	
C10	0.1557 (2)	−0.06397 (19)	0.58621 (18)	0.0429 (5)	
C11	0.17917 (18)	0.28366 (18)	0.46017 (19)	0.0419 (5)	

### Atomic displacement parameters (Å^2^)

**Table:**

	*U*^11^	*U*^22^	*U*^33^	*U*^12^	*U*^13^	*U*^23^
Ni1	0.0411 (2)	0.01267 (17)	0.02797 (19)	−0.00048 (13)	0.00315 (14)	0.00009 (12)
Cl1	0.0830 (5)	0.0554 (4)	0.0687 (4)	0.0079 (3)	0.0195 (4)	0.0253 (3)
N1	0.0420 (9)	0.0166 (7)	0.0319 (8)	0.0007 (6)	0.0030 (7)	−0.0009 (6)
N2	0.0538 (12)	0.0589 (12)	0.0347 (10)	−0.0021 (9)	0.0074 (9)	0.0018 (9)
O1	0.0476 (9)	0.0236 (7)	0.0370 (8)	−0.0015 (6)	−0.0024 (7)	0.0019 (6)
O2	0.0462 (9)	0.0351 (8)	0.0340 (7)	−0.0063 (6)	0.0083 (7)	−0.0039 (6)
O3	0.0695 (11)	0.0353 (8)	0.0500 (10)	−0.0096 (7)	0.0104 (8)	−0.0005 (7)
O4	0.0557 (10)	0.0410 (9)	0.0795 (12)	−0.0068 (7)	0.0228 (9)	−0.0210 (8)
O5	0.0731 (13)	0.0458 (10)	0.0537 (10)	0.0071 (8)	0.0020 (10)	0.0065 (8)
O6	0.0751 (14)	0.0873 (14)	0.0481 (10)	0.0270 (10)	0.0226 (10)	0.0193 (9)
C1	0.0597 (14)	0.0203 (9)	0.0305 (10)	−0.0001 (8)	0.0075 (10)	−0.0023 (8)
C2	0.0601 (14)	0.0186 (9)	0.0325 (11)	−0.0011 (8)	0.0082 (10)	0.0023 (8)
C3	0.0325 (10)	0.0169 (9)	0.0346 (10)	0.0011 (7)	0.0023 (8)	0.0008 (7)
C4	0.0578 (13)	0.0201 (9)	0.0308 (10)	0.0006 (8)	0.0061 (9)	−0.0036 (8)
C5	0.0570 (13)	0.0190 (9)	0.0321 (11)	−0.0002 (8)	0.0057 (9)	0.0042 (8)
C6	0.0457 (13)	0.0504 (14)	0.0410 (12)	−0.0061 (10)	0.0055 (10)	−0.0107 (10)
C7	0.0318 (11)	0.0374 (11)	0.0376 (11)	−0.0035 (8)	0.0039 (9)	−0.0055 (9)
C8	0.0526 (14)	0.0384 (12)	0.0340 (11)	−0.0028 (10)	0.0081 (10)	−0.0004 (9)
C9	0.0543 (14)	0.0353 (11)	0.0432 (13)	0.0000 (10)	0.0094 (11)	−0.0036 (10)
C10	0.0408 (13)	0.0447 (13)	0.0440 (13)	0.0027 (9)	0.0080 (10)	0.0089 (10)
C11	0.0326 (12)	0.0358 (11)	0.0570 (14)	−0.0041 (9)	0.0032 (10)	−0.0103 (10)

### Geometric parameters (Å, º)

**Table:**

Ni1—O1^i^	2.0643 (15)	O6—H61	0.818 (10)
Ni1—O1	2.0643 (15)	O6—H62	0.813 (10)
Ni1—N1^i^	2.0715 (14)	C1—C2	1.375 (3)
Ni1—N1	2.0715 (14)	C1—H1	0.9300
Ni1—O2^i^	2.0850 (13)	C2—C3	1.385 (2)
Ni1—O2	2.0850 (13)	C2—H2	0.9300
Cl1—C10	1.741 (2)	C3—C4	1.384 (2)
N1—C1	1.333 (2)	C3—C3^ii^	1.483 (3)
N1—C5	1.334 (2)	C4—C5	1.376 (3)
N2—C10	1.319 (3)	C4—H4	0.9300
N2—C6	1.337 (3)	C5—H5	0.9300
O1—H11	0.811 (9)	C6—C7	1.378 (3)
O1—H12	0.818 (9)	C6—H6	0.9300
O2—H21	0.813 (9)	C7—C8	1.383 (3)
O2—H22	0.807 (10)	C7—C11	1.513 (3)
O3—C11	1.248 (2)	C8—C9	1.374 (3)
O4—C11	1.257 (2)	C8—H8	0.9300
O5—H51	0.822 (10)	C9—C10	1.375 (3)
O5—H52	0.815 (10)	C9—H9	0.9300
			
O1^i^—Ni1—O1	180.0	C1—C2—C3	119.92 (17)
O1^i^—Ni1—N1^i^	89.62 (6)	C1—C2—H2	120.0
O1—Ni1—N1^i^	90.37 (6)	C3—C2—H2	120.0
O1^i^—Ni1—N1	90.38 (6)	C4—C3—C2	116.48 (16)
O1—Ni1—N1	89.62 (6)	C4—C3—C3^ii^	121.39 (19)
N1^i^—Ni1—N1	180.0	C2—C3—C3^ii^	122.13 (19)
O1^i^—Ni1—O2^i^	90.61 (6)	C5—C4—C3	120.04 (17)
O1—Ni1—O2^i^	89.39 (6)	C5—C4—H4	120.0
N1^i^—Ni1—O2^i^	89.00 (5)	C3—C4—H4	120.0
N1—Ni1—O2^i^	91.00 (5)	N1—C5—C4	123.37 (17)
O1^i^—Ni1—O2	89.39 (6)	N1—C5—H5	118.3
O1—Ni1—O2	90.61 (6)	C4—C5—H5	118.3
N1^i^—Ni1—O2	91.00 (5)	N2—C6—C7	124.1 (2)
N1—Ni1—O2	89.00 (5)	N2—C6—H6	117.9
O2^i^—Ni1—O2	180.0	C7—C6—H6	117.9
C1—N1—C5	116.61 (16)	C6—C7—C8	117.23 (19)
C1—N1—Ni1	122.63 (12)	C6—C7—C11	121.10 (18)
C5—N1—Ni1	120.76 (12)	C8—C7—C11	121.65 (18)
C10—N2—C6	116.23 (18)	C9—C8—C7	120.18 (19)
Ni1—O1—H11	114.7 (16)	C9—C8—H8	119.9
Ni1—O1—H12	115.0 (16)	C7—C8—H8	119.9
H11—O1—H12	102 (2)	C8—C9—C10	117.0 (2)
Ni1—O2—H21	127.6 (17)	C8—C9—H9	121.5
Ni1—O2—H22	117.6 (16)	C10—C9—H9	121.5
H21—O2—H22	111 (2)	N2—C10—C9	125.3 (2)
H51—O5—H52	113 (3)	N2—C10—Cl1	116.40 (16)
H61—O6—H62	105 (3)	C9—C10—Cl1	118.34 (17)
N1—C1—C2	123.56 (17)	O3—C11—O4	124.1 (2)
N1—C1—H1	118.2	O3—C11—C7	118.15 (17)
C2—C1—H1	118.2	O4—C11—C7	117.69 (19)
			
C5—N1—C1—C2	1.2 (3)	N2—C6—C7—C11	−176.9 (2)
Ni1—N1—C1—C2	−177.83 (16)	C6—C7—C8—C9	−1.2 (3)
N1—C1—C2—C3	0.0 (3)	C11—C7—C8—C9	177.10 (19)
C1—C2—C3—C4	−1.1 (3)	C7—C8—C9—C10	0.5 (3)
C1—C2—C3—C3^ii^	178.6 (2)	C6—N2—C10—C9	0.0 (3)
C2—C3—C4—C5	1.0 (3)	C6—N2—C10—Cl1	179.70 (16)
C3^ii^—C3—C4—C5	−178.7 (2)	C8—C9—C10—N2	0.1 (3)
C1—N1—C5—C4	−1.3 (3)	C8—C9—C10—Cl1	−179.55 (16)
Ni1—N1—C5—C4	177.75 (16)	C6—C7—C11—O3	166.0 (2)
C3—C4—C5—N1	0.2 (3)	C8—C7—C11—O3	−12.3 (3)
C10—N2—C6—C7	−0.8 (3)	C6—C7—C11—O4	−12.0 (3)
N2—C6—C7—C8	1.4 (3)	C8—C7—C11—O4	169.7 (2)

Symmetry codes: (i) −*x*+1, −*y*+1, −*z*+1; (ii) −*x*+1, −*y*+2, −*z*+1.

### Hydrogen-bond geometry (Å, º)

**Table:**

*D*—H···*A*	*D*—H	H···*A*	*D*···*A*	*D*—H···*A*
O1—H11···O3^i^	0.81 (1)	1.95 (1)	2.756 (2)	175 (2)
O1—H12···O5	0.82 (1)	1.90 (1)	2.715 (2)	175 (2)
O2—H21···N2^iii^	0.81 (1)	2.08 (1)	2.885 (2)	172 (2)
O2—H22···O4	0.81 (1)	1.96 (1)	2.757 (2)	169 (2)
O5—H51···O3^iv^	0.82 (1)	1.96 (1)	2.776 (2)	172 (3)
O5—H52···O6^v^	0.82 (1)	2.01 (1)	2.790 (3)	160 (3)
O6—H61···O4	0.82 (1)	1.94 (1)	2.753 (2)	177 (3)
O6—H62···O4^vi^	0.81 (1)	2.23 (1)	3.035 (3)	174 (3)
C4—H4···O6^iii^	0.93	2.40	3.288 (3)	160
C9—H9···O5^vii^	0.93	2.53	3.447 (3)	169

Symmetry codes: (i) −*x*+1, −*y*+1, −*z*+1; (iii) −*x*+1/2, *y*+1/2, −*z*+3/2; (iv) *x*+1/2, −*y*+1/2, *z*+1/2; (v) *x*+1, *y*, *z*; (vi) −*x*, −*y*+1, −*z*+1; (vii) −*x*+1, −*y*, −*z*+1.
