# Supplementary material for: Synthesis and crystal structure of a 6-chloro­nicotinate salt of a one-dimensional cationic nickel(II) coordination polymer with 4,4′-bi­pyridine
Source: Acta Crystallogr E Crystallogr Commun. 2020 Apr 2;76(Pt 5):599–604. doi: 10.1107/S2056989020004193 (PMC7199272; doi:10.1107/S2056989020004193)
Supplement: Supplementary file 3 [file e-76-00599-sup3.docx]

**Synthesis and crystal structure of a 6-chloronicotinate salt of a one-dimensional cationic nickel(II) coordination polymer with 4,4'-bipyridine**

**Nives Politeo,**^a^ **Mateja Pisa**č**i**ć**,**^b^ **Marijana** Đ**akovi**ć**,**^b^ **Vesna Sokol**^a^*** and Boris-Marko Kukovec**^a^

**^a^**Department of Physical Chemistry, Faculty of Chemistry and Technology, University of Split, Ruđera Boškovića 35, HR-21000 Split, Croatia, and **^b^**Department of Chemistry, Faculty of Science, University of Zagreb, Horvatovac, 102a, HR-10000 Zagreb, Croatia

Correspondence email: vsokol@ktf-split.hr

**Click the grey 'Authors' label above or use the 'IUCr authors' toolbar button to edit the authors**

Supporting information


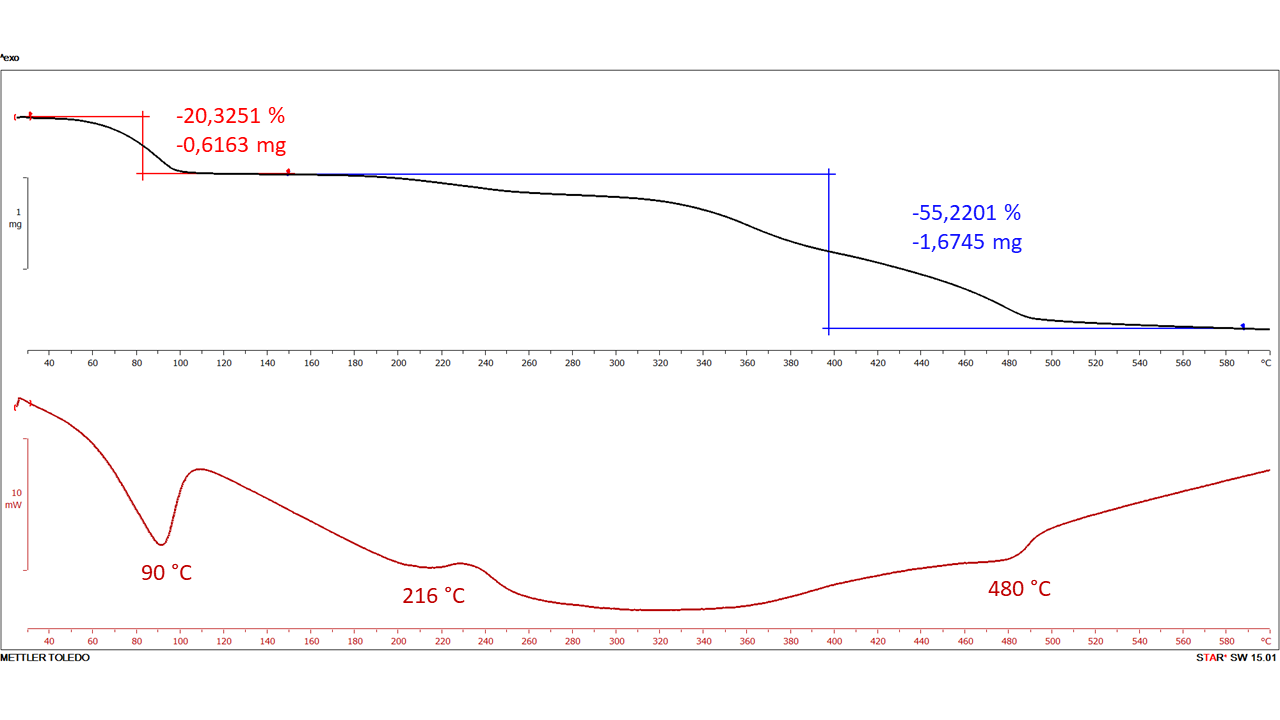


1. TGA (top) and DSC (bottom) curves of **1**.


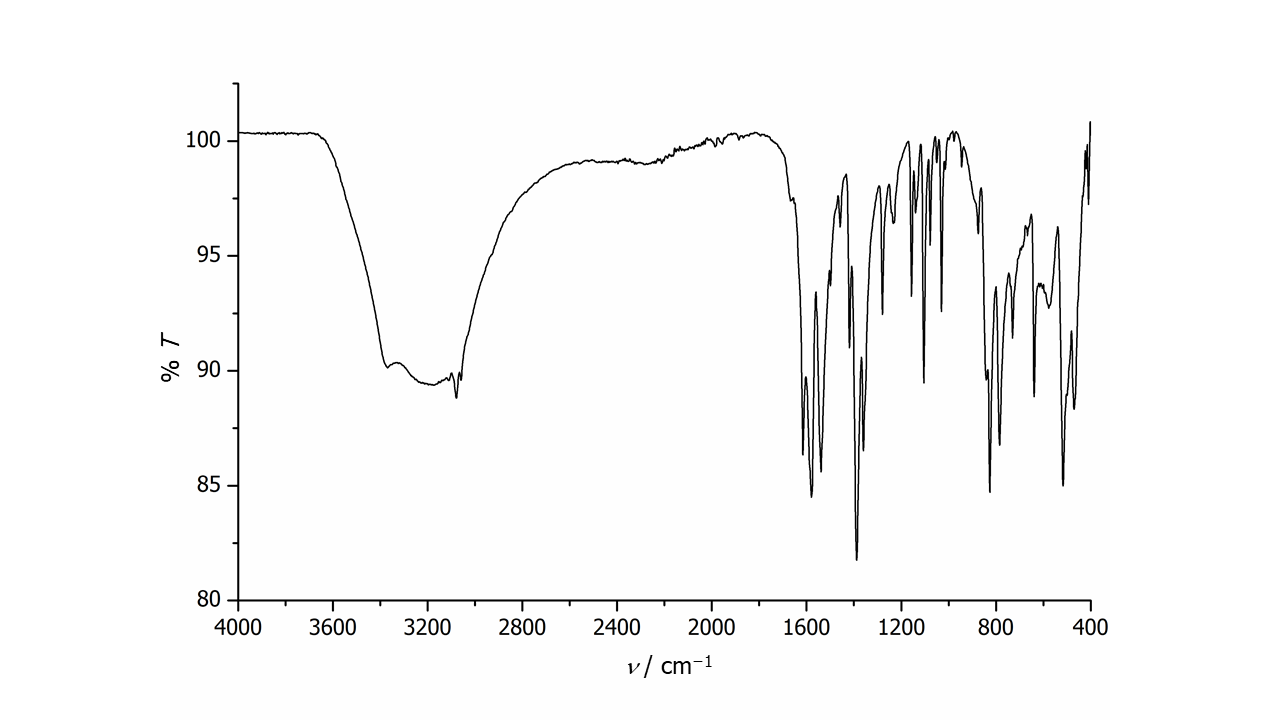


1. IR spectrum of **1**.

**Table S1** IR bands of **1**.

| *ν* (cm^−1^) | %*T* |
| --- | --- |
| 3376 | 88 |
| 3078 | 87 |
| 3059 | 87 |
| 1615 | 81 |
| 1579 | 78 |
| 1539 | 87 |
| 1419 | 88 |
| 1388 | 72 |
| 1360 | 80 |
| 1280 | 90 |
| 1229 | 95 |
| 1157 | 90 |
| 1105 | 84 |
| 1078 | 93 |
| 1030 | 89 |
| 875 | 94 |
| 826 | 77 |
| 785 | 80 |
| 730 | 87 |
| 639 | 81 |
| 517 | 75 |
| 469 | 84 |
| 410 | 94 |
